# Supplementary material for: Appropriate Lymph Node Dissection Sites for Cancer in the Body and Tail of the Pancreas: A Multicenter Retrospective Study
Source: Cancers (Basel). 2022 Sep 11;14(18):4409. doi: 10.3390/cancers14184409 (PMC9497245; doi:10.3390/cancers14184409)
Supplement: Supplementary file 1 [file cancers-14-04409-s001.zip › cancers-1890744-supplementary.pdf]

Table S1. Distribution of lymph node metastasis based on tumor site and efficacy index according to the 2-year disease-free survival rate.

	Total patients n = 235	Pb n=109			Pbt n=51			Pt n=75		
	Frequency of metastasis	Frequency of metastasis	2-year DFS rate	2-year DFS index	Frequency of metastasis	2-year DFS rate	2-year DFS index	Frequency of metastasis	2-year DFS rate	2-year DFS index
#8a/p	18 (7.7%)	12 (11.0%)	36.36	4.00	4 (7.8%)	75.00	5.85	2 (2.7%)	0.00	0.0
#10	16 (6.8%)	0	N.A.	N.A.	4 (7.8%)	0.00	0.0	12 (16.0%)	58.33	9.33
#11d	47 (20.0%)	5 (4.6%)	20.00	0.92	14 (27.5%)	15.38	4.23	28 (37.3%)	33.33	12.43
#11p	62 (26.4%)	39 (35.8%)	32.43	11.61	13 (25.5%)	33.85	8.63	10 (13.3%)	62.50	8.31
#14p	3 (1.3%)	1 (0.9%)	0.00	0.0	1 (2.0%)	0.00	0.0	1 (1.3%)	0.00	0.0
#16b1	5 (2.1%)	2 (1.8%)	0.00	0.0	1 (2.0%)	0.00	0.0	2 (2.7%)	0.00	0.0
#18	37 (15.7%)	17 (15.6%)	18.75	2.93	8 (15.7%)	25.00	3.93	12 (16.0%)	36.36	5.82

Pb, pancreatic body (and/or neck); Pbt, pancreatic body and tail; Pt, pancreatic tail; DFS, disease-free survival; #8a/p, lymph nodes (LNs) along the common hepatic artery; #10, LNs at the splenic hilum; #11d, LNs along the distal splenic artery; #11p, LNs along the proximal splenic artery; #14p, LNs along the proximal superior mesenteric artery; #16b1, LNs around the abdominal aorta; #18, LNs along the inferior margin of the pancreas; N.A., not applicable
